# Absence of mutations in the ATM gene in forty-seven cases of sporadic breast cancer

**DOI:** 10.1038/sj.bjc.6690630

**Published:** 1999-08

**Authors:** D G Bebb, Z Yu, J Chen, M Telatar, K Gelmon, N Phillips, R A Gatti, B W Glickman

**Affiliations:** Centre for Environmental Health, Department of Biology, University of Victoria, P.O. Box 3020, Victoria, BC, V8W 3N5, Canada; Departments of Advanced Therapeutics and Cancer Control Strategy, British Columbia Cancer Research Centre, 601 W. 10th Avenue, Vancouver, BC, V5Z 1L3, Canada; Department of Pathology and Laboratory Medicine, University of British Columbia, 2211 Wesbrook Mall, Vancouver, BC, V6T 2B5, Canada; Department of Pathology, School of Medicine, University of California, Los Angeles, CA, 90095 –1732, USA

**Keywords:** breast cancer, ataxia telangiectasia, protein truncation test (PTT), cancer predisposition

## Abstract

Epidemiological evidence points to an increased risk of breast cancer in ataxia telangiectasia (AT) heterozygote women. Previous attempts to screen early onset or familial breast cancer patients failed to confirm an association. The issue of AT and late onset sporadic breast cancer remained unresolved. We screened 47 women who developed later onset, sporadic breast cancer for ataxia telangiectasia mutated (ATM) mutations. No mutations were found. © 1999 Cancer Research Campaign


*British Journal of Cancer* (1999) **80**(12), 1979–1981
© 1999 Cancer Research Campaign
Article no. bjoc.1999.0630

### Absence of mutations in the ATM gene in forty-seven cases of sporadic breast cancer

**DG Bebb[1,2,4], Z Yu[1], J Chen[1], M Telatar[5], K Gelmon[2], N Phillips[3], RA Gatti[5] and BW Glickman[1]**

[1]Centre for Environmental Health, Department of Biology, University of Victoria, P.O. Box 3020, Victoria, BC V8W 3N5, Canada; Departments of [2]Advanced Therapeutics and [3]Cancer Control Strategy, British Columbia Cancer Research Centre, 601 W. 10th Avenue, Vancouver, BC V5Z 1L3, Canada; [4]Department of Pathology and Laboratory Medicine, University of British Columbia, 2211 Wesbrook Mall, Vancouver, BC V6T 2B5, Canada; [5]Department of Pathology, School of Medicine, University of California, Los Angeles, CA90095 –1732, USA

**Summary** Epidemiological evidence points to an increased risk of breast cancer in ataxia telangiectasia (AT) heterozygote women. Previous attempts to screen early onset or familial breast cancer patients failed to confirm an association. The issue of AT and late onset sporadic breast cancer remained unresolved. We screened 47 women who developed later onset, sporadic breast cancer for ataxia telangiectasia mutated (ATM) mutations. No mutations were found.

**Keywords**: breast cancer; ataxia telangiectasia; protein truncation test (PTT); cancer predisposition

A hereditary component to breast cancer was first described by the Romans and clearly documented by Broca, the French surgeon, over a hundred years ago (Broca, 1866). Although three genes (p53, Brcal and Brca2) are now known to play a role in hereditary breast cancer (Ford and Easton, 1995), numerous families exist that carry no identifiable mutations in any of these genes in which breast cancer occurs much more often than would be expected by chance (Lynch et al, 1976; Ford and Easton, 1995). Clearly, additional heritable factors, perhaps less penetrant than Brcal, Brca2 and p53, play an important, but as yet poorly understood, part in breast cancer aetiology (Serova et al, 1997).

In 1976, Swift published data that suggested an increased relative risk of developing breast cancer among the members of ataxia telangiectasia (AT) families. AT can be regarded as an inherited cancer predisposition syndrome (Gatti, 1998) and there is evidence to suggest that the cancer predisposition extends to AT heterozygotes, although the magnitude and nature of the predisposition is controversial (Easton, 1994). The absence of clinical manifestation, and the lack of quantifiable in vitro cellular characteristics make identifying AT carriers in the general population unreliable (Heim et al, 1992; Scott et al, 1993; Bebb et al, 1998). Unfortunately, the size of the gene (150 Kb genomic, 13 Kb cDNA, 66 exons) and the lack of mutational hot spots makes screening approaches to mutation detection unwieldy to date (Savitsky et al, 1995; Gilad et al, 1996).

Analysis of the mutational spectrum of the ataxia telangiectasia mutated (ATM) gene reveals that a large majority of mutations yield transcripts that would result in truncated protein products (Gilad et al, 1996; Telatar et al, 1996) which can be detected by the protein truncation test (PTT). This approach was used to screen a large number of women who had developed breast cancer before the age of forty for mutations in the ATM gene (Fitzgerald et al, 1997). Out of 401 cases, only two mutations were found, a similar incidence to that found in the control group. More recently, Chen et al (1998), using the same methodology, failed to confirm an association between ATM mutations and familial breast cancer.

In contrast, Athma et al (1996) have suggested that although ATM is important in breast cancer, it is likely manifest only in the older population of breast cancer patients. Here we describe an attempt to investigate the frequency of ATM mutations in a population of ‘sporadic’ breast cancer patients of a slightly older age group than in the Fitzgerald study. Forty-seven women diagnosed with breast cancer were screened for ATM mutations using the PTT; no mutations were found.

*Received 24 August 1998*
*Revised 2 February 1999*
*Accepted 16 February 1999*

*Correspondence to:* W Glickman

### MATERIALS AND METHODS

### Patient selection

A series of forty-seven patients were recruited from weekly breast cancer clinics at the British Columbia Cancer Agency (BCCA), Vancouver, Canada. Consent for genetic studies was obtained by a physician using approved consent forms drawn up specifically for the study. None of the patients were being investigated for high-density familial breast or ovarian cancer.

### Blood sampling and RNA extraction

Ten millilitres of peripheral blood were obtained from the donors by venipuncture and collected in leucoprep (Becton Dickenson) tubes. Total RNA was extracted from the buffy coat by a guanidium thiocyanate–phenol–chloroform single step reaction using RNA extraction kits (“RNeasy”, Qiagen, California).

### cDNA generation

First-strand cDNA was prepared in two separate 25 μl reactions using a total of four reverse primers. Each reaction, in addition to the appropriate primers, contained 1 µg total RNA, 1 × first-strand buffer, 20 units RNAase inhibitor, 10 mM dithiothreitol, 3 mM dNTP and 100 units of M-MLV reverse transcriptase. The reaction mixture was incubated for 1 h at 37°C and 5 µl of the reaction product used as a polymerase chain reaction (PCR) template.

### Primers and PCR

As previously described (Telatar et al, 1998), the ATM gene was divided into seven overlapping regions: a (1387 bp), b (1247 bp), c (1534 p), d (1521 bp), e (1316 bp), f (1769 bp) and g (1655 bp). Primers were designed to include the T7 promoter sequence for the initiation of transcription by T7 RNA polymerase. PCR of each region was performed.

### Protein truncation test

A total of 100 ng rt-PCR product was used directly as template for the coupled in vitro transcription translation reaction using rabbit reticulocyte lysate according to the manufacturer's (Promega) recommended protocol. Reactions were performed in a 12.5 µl total volume with 6 µci of 35S-methionine as label. Products were separated by means of 14% discontinuous SDS-PAGE at 200 volts for 3 h. The gel was then soaked in Amplify (Amersham) for 30 min, dried and placed on X-ray film for between 6 and 48 h.

### RESULTS

### Patient details

Forty-seven women diagnosed with breast cancer were enrolled in the study. Their age at diagnosis ranged from 30 to 78, mean age at diagnosis was 53.4 years. All but four were aged over 40, and 29 of the patients fell in the age group between 40 and 59. The group included mainly invasive ductal carcinoma of the breast. A variety of treatment modalities, including radiation, were used in the management of these patients. In all 47 samples screened, no truncated products were detected. The PTT was successful in identifying a mutation in the positive control, a known AT heterozygote (parent of an affected individual).

### DISCUSSION

Our results do not support the hypothesis that carriers of the ataxia telangiectasia gene make up a significant proportion of the breast cancer population. However, neither do our results exclude the possibility that inheriting the ATM gene predisposes women to breast cancer (Tables 1 and 2). Several additional factors may influence the significance of the results.

First of all, the exact magnitude of the relative risk estimated for developing breast cancer in female AT carriers and the proportion of breast cancer cases attributable to AT heterozygosity is unclear. Swift's initial estimate (1976) was undermined by the low incidence of breast cancer observed in his spouse control population (Easton, 1994). His subsequent 6-year prospective analysis of cancer incidence in 161 AT families identified a 5.1-fold increased risk of breast cancer in female AT heterozygotes (Swift et al, 1991). Other investigators confirmed an elevated risk of breast cancer in AT carriers (Pippard et al, 1988; Borresen et al, 1990) but none documented a relative risk as high as Swift's estimates. Also uncertain is the manner in which such a predisposition would

**Table 1** Probability of finding no ATM mutations under different statistical conditions: women of all ages

| PTT sensitivity | Breast cancer population made up of AT carriers (%) | The chance of observing 0 mutations in 47 cases |
|---|---|---|
| 99 | 5 | 0.09 |
| 99 | 10 | 0.007 |
| 99 | 15 | 0.0005 |
| 70 | 5 | 0.19 |
| 70 | 10 | 0.03 |
| 70 | 15 | 0.005 |

**Table 2** Probability of finding no ATM mutations under different statistical conditions: women aged over 40

| PTT sensitivity | Breast cancer population made up of AT carriers (%) | The chance of observing 0 mutations in 47 cases |
|---|---|---|
| 99 | 5 | 0.113 |
| 99 | 10 | 0.011 |
| 99 | 15 | 0.001 |
| 70 | 5 | 0.22 |
| 70 | 10 | 0.04 |
| 70 | 15 | 0.008 |

Using the following equation***:

$$P = {}_0\Sigma^{47}\ (1\text{-sensitivity})^n\ \text{prevalence}^n\ (1\text{-prevalence})^{47-n}\ \frac{47!}{n!(47-n)!}$$

[1]Where n is the true number of ATM mutations. **It is assumed that specificity of PTT is 100%

affect the age pattern of breast cancer incidence. It may be, as suggested by Athma et al (1996), that the ATM gene acts more like a hereditary susceptibility factor rather than a highly penetrant gene, becoming manifest only in an older population (Kinzler and Vogelstein, 1997). Another malignancy in which ATM heterozygosity may play a role, namely T-cell pro-lymphocytic leukaemia (T-PLL) (Vorechovsky et al, 1997; Yuille et al, 1998) has an average age at diagnosis of 69 years.

A third factor to consider is the efficiency of the PTT assay in detecting ATM mutations. Initial estimates that 95% or more ATM mutations would result in a truncated product (Gilad et al, 1996) were later tempered to roughly 70% (Telatar et al, 1996; Chen et al, 1998; Stankovic et al, 1998). If that is so, applying the PTT assay for screening purposes will miss 30% of mutations despite its very high efficiency as a test. On the other hand, in our experience, PTT identifies ATM mutations in the parents of AT patients with equal efficiency (RA Gatti et al, unpublished data). Finally, the frequency of the AT gene in the population is not clear. Estimates of the incidence of AT vary between 1:40 000 (USA) and 1:100 000 (UK) live births. Whether the gene frequency is relatively constant worldwide or even in North America, is not certain.

Consequently, the statistical power of our study ranges in significance depending on how these variables are put together. On the basis of our study assuming a PTT sensitivity of 99%, it is improbable that AT heterozygotes make up 6.2% or more of the sporadic breast cancer population or 6.8% of the breast cancer population aged over 40 at diagnosis ($P = 0.05$). Even with a PTT sensitivity of only 70%, it is unlikely that AT heterozygotes make up more than 8.8% of the breast cancer population in general or 9.6% of the

 *© 1999 Cancer Research Campaign*

breast cancer population over 40 at diagnosis (*P* = 0.05) (Tables 1 and 2). The sample size in our study is sufficient to detect a six- to ninefold increase of prevalence of AT heterozygotes among the population of late onset sporadic breast cancer over the normal level with 80% power.

The epidemiological association between heterozygosity for AT and breast cancer has intrigued oncologists and tumour biologists for over 20 years. The issue is clearly important and requires clarification. Confirming the association would help account for the increased incidence of breast cancer in women irradiated for Hodgkin's disease (Bhatia et al, 1996) and support suggestions of a radiosensitive subgroup among breast cancer patients (Norman et al, 1992; Lavin et al, 1994; Scott et al, 1998). It would confirm that DNA repair and processing deficiencies, already implicated in the aetiology of colon cancer (Lynch et al, 1997), have a role in breast carcinogenesis as well (Scully et al, 1997; Sharan et al, 1997). Our data suggest that AT heterozygotes do not make up more than 8.8% of the female population who develop sporadic breast cancer but we are unable to discount the possibility that they make up 6.2% or less of the breast cancer population. Multiple smaller scale screening for unique mutations directed at specific ethnic groups may be required to further assess the role of ATM in breast cancer (Telatar et al, 1998).

### REFERENCES

Athma P, Rappaport R and Swift M (1996) Molecular genotyping shows that ataxia-telangiectasia heterozygotes are predisposed to breast cancer. *Cancer Genet Cytogenet* **92**: 130–134

Bebb G, Steele PP, Warrington PJ, Moffat JA and Glickman BW (1998) Caffeine does not potentiate radiation induced DNA damage in ataxia-telangiectasia lymphoblastoid cells. *Mutat Res* **401**: 27–32

Bhatia S, Robison LL, Oberlin O, Greenberg M, Bunin G, Fossati-Bellani F and Medows AT (1996) Breast and other second neoplasms after childhood Hodgkin's disease. *N Engl J Med* **334**: 745–751

Borresen AL, Anderson TI, Treti S, Heiberg A and Moller P (1990) Breast cancer and other cancers in Norwegian families with ataxia-telangiectasia. *Genes Chromosomes Cancer* **2**: 339–340

Broca PP (1866) *Traites des tumeurs* **1**: 80

Chen J, Birkholtz GG, Lindblom P, Rubio C and Lindblom A (1998) The role of ataxia-telangiectasia in familial breast cancer. *Cancer Res* **58**: 1376–1379

Easton DF (1994) Cancer risks in A-T heterozygotes. *Int J Radiat Biol* **66**: S177–182

Fitzgerald MG, Bean JM, Hegde SR, Unsal H, MacDonald DJ, Harkin DP, Finkelstein DM, Isselbacher KJ, and Haber DA (1997) Heterozygous ATM mutations do not contribute to early onset of breast cancer. *Nat Genet* **15**: 307–310

Ford D and Easton DF (1995) The genetics of breast and ovarian cancer. *Br J Cancer* **72**: 805–812

Gatti RA (1998) Ataxia telangiectasis. In: *The Genetic Basis of Human Cancers*, Vogelstein B and Kingley KW (eds.), pp. 275–300, McGraw-Hill: New York

Gilad S, Khosravi R, Shkedy D, Uziel T, Ziv Y, Savitsky K, Rotman G, Smith S, Chessa L, Jorgensen TJ, Harnik R, Frydman M, Sanal O, Portnoi S, Goldwicz Z, Jaspers NGJ, Gatti R, Lenoir G, Lavin M, Tatsumi K, Wegner RD, Shiloh Y and Bar-Shira A (1996) Predominance of null mutations in ataxia-telangiectasia. *Hum Mol Genet* **5**: 433–439

Heim RA, Lench NJ and Swift M (1992) Heterozygous manifestations in four autosomal recessive cancer-prone syndromes, ataxia-telangiectasia, xeroderma pigmentosum, Fanconi anemia and Bloom syndrome. *Mutat Res* **284**: 25–36

Kinzler KW and Vogelstein B (1997) Gatekeepers and caretakers. *Nature* **386**: 761–762

Lavin MF, Bennett I, Ramsay J, Gardiner RA, Seymor GJ, Farrell A and Walsh M (1994) Identification of a potentially sensitive subgroup among patients with breast cancer. *J Natl Cancer Inst* **86**: 1627–1634

Lynch HT, Mulchahy GM and Lynch P (1976) Genetic factors in breast cancer, a survey. *Pathol Ann* **11**: 77–101

Lynch HT, Smyrk T and Lynch J (1997) An update of HNPCC, Lynch syndrome. *Cancer Genet Cytogenet* **93**: 84–99

Norman A, Iwamoto KS, Kagan AR and Wollin M (1992) Radiation sensitive breast cancer patients. *Radiother Oncol* **23**: 196–197

Pippard EC, Hall AJ, Barker DJ and Bridges BA (1988) Cancer in homozygotes and heterozygotes of ataxia-telangiectasia and xeroderma pigmentosum in Britain. *Cancer Res* **48**: 2929–2932

Savitsky K, Bar-Shira A, Gilad S, Rotman G, Ziv Y, Vanagaite L, Tagle DA, Smith S, Uziel T, Sfez S, Ashkenazi M, Pecker I, Frydman M, Harnik R, Patanjali SR, Simmons A, Clines GA, Sartiel A, Gatti RA, Chessa L, Sanal O, Lavin MF, Jaspers NGJ, Taylor AMR, Arlett CF, Miki T, Weissman SM, Lovett M, Collins FS and Shiloh Y (1995) A single ataxia-telangiectasia gene with a product similar to PI-3 kinase. *Science* **268**: 1749–1753

Scott D, Jones LA, Elyan SAG, Spreadborough A, Cowan R and Ribiero G (1993) Identification of AT-heterozygotes. In: *Ataxia-Telangiectasia*, Gatti RA and Palmer RB (eds.), pp. 101–116. NATO ASI Series vol H 77

Scott D, Barber JBP, Levine EL, Burrill W and Roberts SA (1998) Radiation-induced micronucleus induction in lymphocytes identifies a high frequency of radiosensitive cases among breast cancer patients – a test for predisposition. *Br J Cancer* **77**: 614–620

Scully R, Chen J, Plug A, Xiao Y, Weaver D, Feunteun J, Ashley T and Livingston DM (1997) Association of Brcal with Rad51 in mitotic and meiotic cells. *Cell* **88**: 265–275

Serova OM, Mazoyer S, Puget N, Dubois V, Tonin P, Shugart YY, Goldgar D, Narod SA, Lynch HT and Lenoirm GM (1997) Mutations in Brcal and Brca2 in breast cancer families, are there more breast cancer-susceptibility genes? *Am J Hum Genet* **60**(3): 486–495

Sharan SK, Morimatsu M, Albrecht U, Lim DS, Regel E, Dinh C, Sands A, Eichele G, Hasty P and Bradley A (1997) Embryonic lethality and radiation hypersensitivity mediated by Rad51 in mice lacking Brca2. *Nature* **386**: 804–810

Stankovic T, Kidd AMJ, Sutcliffe A, Mcguire GM, Robinson P, Weber P, Bedenham T, Bradwell AR, Easton DF, Lennox GG, Haites N, Byrd PJ and Taylor AMR (1998) ATM mutations and phenotypes in ataxia-telangiectasia families in the British Isles – expression of mutant ATM and the risk of leukemia, lymphoma and breast cancer. *Am J Hum Genet* **62**: 334–345

Swift M, Sholman L, Perry M and Chase C (1976) Malignant neoplasms in the families of patients with ataxia-telangiectasia. *Cancer Res* **36**: 209–215

Swift M, Morrell D, Massey RB and Chase CL (1991) Incidence of cancer in 161 families affected by ataxia-telangiectasia. *N Engl J Med* **325**: 1831–1836

Telatar M, Wang Z, Udar N, Liang T, Bernatowska-Matuszkewicz E, Lavin M, Shiloh Y, Concannon P, Good RA and Gatti RA (1996) Ataxia-telangiectasia, mutations in ATM cDNA detected by protein truncation screening. *Am J Hum Genet* **59**: 40–44

Telatar M, Teraoka S, Wang ZJ, Chun HH, Liang T, Castellvibel S, Udar N, Borresendale AL, Chessa L, Bernatowska-Matuszkiewicz E, Porras O, Watanabe M, Junder A, Concannon P and Gatti RA (1998) Ataxia-telangiectasia – identification and detection of founder-effect mutations in the ATM gene in ethnic populations. *Am J Hum Genet* **62**: 86–97

Telatar M, Wang Z, Castellvi-Bel S, Tai L-Q, Rivero-Carmena M, Regueiro JR, Porras O and Gatti RA (1999) A model for ATM heterozygote identification in a large population, Four founder effect ATM mutations identify most of Costa Rican patients with ataxia-telangiectasia. *Mol Genet Metab* (in press)

Tokunga M, Land CE and Yamamoto T (1987) Incidence of breast cancer among atomic bomb survivors, Hiroshima and Nagasaki, 1950–1980. *Radiat Res* **112**: 243–272

Vorechovsky I, Luo L, Dyer MJS, Catovsky D, Amlot P, Yaxley JC, Foroni L, Hammarstrom L, Webster ADB and Yuille M (1997) Clustering of missense mutations in the ataxia-telangiectasia gene in sporadic T-cell leukaemia. *Nat Genet* **17**: 96–99

Yuille MAR, Coignet LJA, Abraham SM, Yaqub F, Luo L, Matutes E, Britobabapulle V, Vorechovsky I, Dyer MJS and Catovsky D (1998) ATM is usually rearranged in T-cell prolymphocytic leukaemia. *Oncogene* **16**: 789–796

*© 1999 Cancer Research Campaign*
